# High-performance cavity-enhanced quantum memory with warm atomic cell

**DOI:** 10.1038/s41467-022-30077-1

**Published:** 2022-05-02

**Authors:** Lixia Ma, Xing Lei, Jieli Yan, Ruiyang Li, Ting Chai, Zhihui Yan, Xiaojun Jia, Changde Xie, Kunchi Peng

**Affiliations:** 1 State Key Laboratory of Quantum Optics and Quantum Optics Devices, Institute of Opto-Electronics, Shanxi University, Taiyuan, 030006 P. R. China; 2 Collaborative Innovation Center of Extreme Optics, Shanxi University, Taiyuan, 030006 P. R. China

**Keywords:** Quantum optics, Quantum information

## Abstract

High-performance quantum memory for quantized states of light is a prerequisite building block of quantum information technology. Despite great progresses of optical quantum memories based on interactions of light and atoms, physical features of these memories still cannot satisfy requirements for applications in practical quantum information systems, since all of them suffer from trade-off between memory efficiency and excess noise. Here, we report a high-performance cavity-enhanced electromagnetically-induced-transparency memory with warm atomic cell in which a scheme of optimizing the spatial and temporal modes based on the time-reversal approach is applied. The memory efficiency up to 67 ± 1% is directly measured and a noise level close to quantum noise limit is simultaneously reached. It has been experimentally demonstrated that the average fidelities for a set of input coherent states with different phases and amplitudes within a Gaussian distribution have exceeded the classical benchmark fidelities. Thus the realized quantum memory platform has been capable of preserving quantized optical states, and is ready to be applied in quantum information systems, such as distributed quantum logic gates and quantum-enhanced atomic magnetometry.

## Abstract (web summary)

Quantum memories usually suffer from a trade-off between efficiency and excess noise. Here, by exploiting the time-reversal approach for improving modes matching, the authors show a warm-atomic-cell-based cavity-enhanced memory with 67% efficiency and noise level close to quantum noise limit.

## Introduction

The high-performance quantum memory featuring both high memory efficiency and low excess noise is an indispensable building block in quantum information systems, distributed quantum computation and quantum metrology. For example, the multiple spatial separated macroscopic objects can be entangled by efficiently storing multipartite entangled state of optical modes, and its realization depends on memory efficiency and noise level^1^. In distributed quantum computation, enhancing the memory fidelity among entangled different modules (nodes) is significant for implementing optical logical gates^2,3^. It has been demonstrated that the atom-based measurement sensitivity is ultimately restricted by the quantum noise limit (QNL), and spin squeezing holds a promise to overcome this restriction^4,5^. In quantum-enhanced atomic magnetometry, the spin squeezing can be generated by efficiently storing the squeezed optical mode, and then used to measure weak signal merged in the quantum noise^6^.

Over the past decades, various light-atom interactions have been utilized to implement quantum memory, such as electromagnetically induced transparency (EIT)^7–12^, far-off-resonance Raman^13^, quantum non-demolition^14^, Autler–Townes splitting^15^ and photon echo interaction^16^. By applying a gradient magnetic field in an atomic cell operating with a special regime, gradient echo memory provides the storage of coherent pulses containing around one photon with the recall fidelity up to 98%^17^. The high memory efficiency is crucially important for practical quantum information^18,19^. Toward the aim of high efficiency, the coherent optical storage efficiency has reached 92.0 ± 1.5% in an optically dense cold atomic media based on EIT effect; however, the excess noise attached to the signal mode is about 6% higher than the QNL^20^. The memory efficiency in warm atomic cell has also been improved by using optimal input signal mode^21^. Besides, another challenge for the memory of quantized optical states is to suppress the excess noise, which will destroy quantum features of stored states. In the processing of atom-light interaction with a Λ-type energy configuration, the coupling of the control mode on the signal mode transition will induce unwanted four-wave-mixing (FWM) noise, which is the main noise source of quantum memory^22,23^. In near-resonant EIT memory with a moderate atomic number, the interaction for storing quantum states is dominant and the influence of FWM noise is relatively less. The EIT memory in the warm atomic vapor with the noise of 2% higher than the QNL and the completed storage amplitude efficiency of 10% has been reported^24^. It has been proved that EIT memories are able to reach the QNL, which have been applied to preserve the squeezed light with atomic ensemble^25,26^. Alternatively, the optical cavity can enhance the light-atom interaction^27–33^ and suppress the excess noise^34^. Although in a demonstrated cavity-enhanced Raman memory the FWM noise has been effectively suppressed, the memory efficiency is below 10%^22^. So far, the effective realization of quantum memory with both high efficiency and low noise approaching the QNL is still a significant challenge for practical applications.

To accomplish quantum memory with the necessary features of both high memory efficiency and low excess noise, we present an experimental demonstration on a cavity-enhanced EIT memory in a simple warm atomic cell, in which the near-perfect mode matching technique based on time-reversal approach is applied^21^. In this way, not only the FWM noise but also the other noises are actively suppressed, due to off resonance with optical cavity; while the memory interaction is effectively enhanced, by resonating the signal mode in the optical cavity. Thanks to the cavity-enhanced EIT interaction and the near-perfect mode matching, both high memory efficiency up to 67 ± 1% and low noise level close to the QNL are simultaneously obtained by the memory system. Based on both high efficiency and low excess noise at QNL level, the average fidelities of the memory measured on a set of input coherent states with varied phases and amplitudes within a Gaussian distribution have totally exceeded the corresponding classical benchmark fidelities. Thus, the performance of the memory has reached a level higher than any classical memory and is able to store quantum states of light^35,36^.

## Results

### Principle of cavity-enhanced quantum memory

**Fig. 1:**
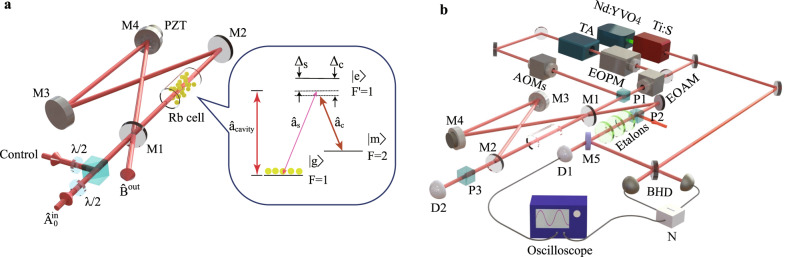
Schematic diagram. **a** Diagram for the cavity-enhanced quantum memory and atomic energy level for quantum memory. Atoms with a ground state $\left|g\right\rangle$: $\left|5{S}_{1/2},F=1\right\rangle$, a meta-stable state $\left|m\right\rangle$: $\left|5{S}_{1/2},F=2\right\rangle$, and an excited state $\left|e\right\rangle$: $\left|5{P}_{1/2},{F}^{\prime}=1\right\rangle$ are shown. The atomic cell is placed between two plano mirrors, where the optical cavity simultaneously enhances the light-atom interaction and suppresses the excess noise. **b** Experimental setup for implementing cavity-enhanced quantum memory. TA tapered amplifier, PZT piezoelectric transducer, EOAM electro-optical amplitude modulator, EOPM electro-optical phase modulator, AOM acousto-optical modulator, BHD balanced homodyne detector, D photoreceiver detector, M mirror, P Glan–Thompson polarizer, N negative power combiner.

In quantum optics, the optical mode is represented by the annihilation operator $$\hat{a}$$, the amplitude (phase) quadrature $${\hat{X}}_{L}$$$$({\hat{Y}}_{L})$$ of light corresponds to the real (imaginary) part of the annihilation operator $$\hat{a}$$, as $${\hat{X}}_{L}=(\hat{a}+{\hat{a}}^{{{{\dagger}}} })/\sqrt{2}$$ ($${\hat{Y}}_{L}=(\hat{a}-{\hat{a}}^{{{{\dagger}}} })/\sqrt{2}i$$)^37^. Under the Holstein–Primakoff approximation, the collective atomic spin wave is described by the lowering operator $$\hat{S}={\sum }_{i}\left|g\right\rangle \left\langle m\right|$$, here $$\left|g\right\rangle$$ and $$\left|m\right\rangle$$ stand for a ground state and meta-stable state, respectively. The amplitude (phase) quadrature $${\hat{X}}_{A}$$$$({\hat{Y}}_{A})$$ of the atoms is associated with the *y*(*z*) component of the Stokes operator $${\hat{S}}_{y}$$$$({\hat{S}}_{z})$$ on the Bloch sphere, which is represented by $$ \hat{X}_{A}=(\hat{S}+{\hat{S}}^{{{{\dagger}}} })/\sqrt{2}={\hat{S}}_{y}/\sqrt{\langle {\hat{S}}_{x}\rangle}$$$$({\hat{Y}}_{A}=({\hat{S}}-{\hat{S}}^{{{{\dagger}}} })/\sqrt{2}i={\hat{S}}_{z}/{\sqrt{\langle {\hat{S}}_{x}\rangle}})$$^14^. The coherent state of light is a minimum uncertainty state with equal uncertainty between two conjugate quadrature components, which is usually used to describe the quantized state of laser. The quantum natures of an optical memory can be characterized by means of preserving coherent state, thus the coherent state of optical mode is utilized as the input state of quantum memory in our experiment. The quantized state can be transferred between light and atomic superposition in the EIT memory^8^. The Λ-type three-level system of a ground state $$\left|g\right\rangle$$, a meta-stable state $$\left|m\right\rangle$$ and an excited state $$\left|e\right\rangle$$ is employed in the EIT configuration, which is presented in the insert of Fig. 1a. The signal mode is near resonant with the transition between a ground state $$\left|g\right\rangle$$ and an excited state $$\left|e\right\rangle$$, while control mode is near resonant with the transition between a meta-stable state $$\left|m\right\rangle$$ and an excited state $$\left|e\right\rangle$$. In our system, the control mode is much stronger than the signal mode, and is treated as a classical mode. When the collective atomic spin wave $$\hat{S}(t)$$ interacts with the signal mode $$\hat{a}(t)$$ via EIT process, the quantized state of the signal mode and the atomic ensemble can be transferred to each other, because the effective Hamiltonian $${\hat{H}}_{EIT}$$ of light-atom interaction is a type of beam-splitter interaction^38^. The quantum memory process includes three stages of writing, storage, and reading that are implemented by modulating the light-atom interaction with a control mode. Therefore, the step-like function used as an approximation of switching on and off processes in EIT interaction can be shown as follows: $$\hat{H}(t)=\hslash \kappa {\hat{a}}^{{{{\dagger}}} }\hat{S}+\; \hslash \kappa {\hat{S}}^{{{{\dagger}}} }\hat{a}$$ (−*∞* < *t* < 0); $$\hat{H}(t)=0$$ (0 < *t* < *T*_0_); $$\hat{H}(t)=\hslash \kappa {\hat{a}}^{{{{\dagger}}} }\hat{S}+\hslash \kappa {\hat{S}}^{{{{\dagger}}} }\hat{a}$$(*T*_0_ < *t* < *∞*), where *T*_0_ is the storage time, $$\kappa =\sqrt{{N}_{a}}\mu {{\Omega }}/{{\Delta }}$$ is the effective light-atom interaction constant, *N*_*a*_ is the atomic number, *μ* is the light-atom coupling constant, Ω is the Rabi frequency of the control mode, and Δ is the detuning between light and atom coupling.

Figure 1a is a diagram for the cavity-enhanced quantum memory with a warm atomic cell. The cavity with a bow-tie-type ring configuration consists of two plano mirrors and two concave mirrors, which enables to enhance the light-atom interaction and suppress the excess noise. The input signal mode $\hat{A}{(t)}^{in}$ is coupled into the cavity mode $\hat{a}$ through the input–output mirror with the coupling rate to the cavity of input mode *γ*_1_ = *T*/(2*τ*), where *T* is the transmission of input–output mirror and *τ* is the round-trip time of the optical mode inside optical cavity. The other three cavity mirrors are highly reflective for the optical signal mode, and one of them is mounted on piezoelectric transducer for scanning or locking the cavity length. The cavity loss *L* is unavoidable in real experiment due to the imperfect coating, and the corresponding decay rate of cavity loss is *γ*_2_ = *L*/(2*τ*), which introduces the vacuum noise $\hat{A}{(t)}_{\upsilon }^{in}$. The atomic spin wave decoherence rate is *γ*_0_, and couples the noise of atomic medium $\hat{S}{(t)}_{\upsilon }$ into cavity mode $\hat{a}$. When the input signal mode resonates with cavity mode and the control mode is near-resonance with the cavity mode, the FWM noise is off-resonance and effectively suppressed. Quantum Langevin equations, describing evolution of observable operators for the cavity mode $\hat{a}(t)$ and collective atomic spin wave $\hat{S}(t)$ are shown as

1 $$\frac{d\hat{a}(t)}{dt}=-\gamma \hat{a}(t)-i\kappa (t)\hat{S}(t)+\sqrt{2{\gamma }_{1}}\hat{A}{(t)}^{in}+\sqrt{2{\gamma }_{2}}\hat{A}{(t)}_{\upsilon }^{in},$$

2 $$\frac{d\hat{S}(t)}{dt}=-{\gamma }_{0}\hat{S}(t)-i\kappa (t)\hat{a}(t)+\sqrt{2{\gamma }_{0}}\hat{S}{(t)}_{\upsilon },$$

where *γ* = *γ*_1_ + *γ*_2_ corresponds the sum of the coupling rate and the decay rate of cavity. For an input signal mode to be stored, a complete mode expansion into the longitudinal modes of the input optical mode is expressed by $\hat{A}{(t)}^{in}=u{(t)}_{0}^{in}{\hat{a}}_{0}^{in}$, where ${\hat{a}}_{0}^{in}$ is an optical mode operator and $u{(t)}_{0}^{in}$ is a temporal mode function of the input optical mode, which determines the optical mode shape. The input mode is dynamically shaped in time to provide optimum memory efficiency, and the temporal mode function in our system is approximately described by a rising exponential function^39^. In the cavity-enhanced memory system, the memory efficiency is defined as the ratio of the photon number of the released signal mode to that of the input signal mode, which depends on storage mechanism, media property and systematic losses. By solving quantum Langevin equations with the proper input temporal mode function, the memory efficiency *η*(*T*_0_) at the storage time *T*_0_ from input optical mode to released optical mode is given by^40^

3 $$\eta ({T}_{0})=\frac{{(-{\gamma }_{1}{\gamma }_{0}^{2}{e}^{-\gamma {T}_{0}}+{\gamma }_{1}{\kappa }^{2}{e}^{-{\gamma }_{0}{T}_{0}})}^{2}}{{({\gamma }_{0}+\gamma )}^{2}{({\kappa }^{2}+{\gamma }_{0}\gamma )}^{2}}.$$

### Experimental realization of cavity-enhanced quantum memory

**Fig. 2:**
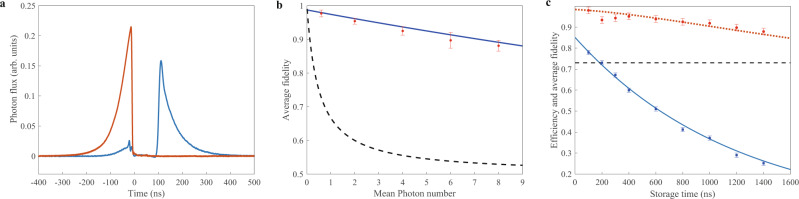
Experimental results. **a** Temporal variation of the experimentally measured photon fluxes. The red line and the blue line indicate the photon fluxes of the input signal mode and the released signal mode from the cavity-enhanced quantum memory system, respectively. **b** The dependence of average fidelity on the mean photon number of the Gaussian distribution set of input coherent states. Blue solid line represents the theoretical average fidelity, and the red dots are the corresponding experimentally measured results. The black dashed line is the corresponding classical benchmark fidelity. **c** The results for memory efficiency and average fidelity vs. storage time. Blue solid line and blue squares are the theoretical memory efficiency and the experimentally measured values, respectively; and red dotted line and red dots represent the theoretical average fidelity and experimentally measured results, respectively; the black dashed line stands for the classical benchmark fidelity. Error bars represent ±1 standard error are obtained with the statistics of the measured photon numbers and noises.

Figure 2a shows the experimentally measured photon fluxes of the input and released signal modes at the storage time of 100 ns, when the atomic cell is heated to around 95 °C. The red line and the blue line indicate the photon fluxes of the input signal mode and the released signal mode from the cavity-enhanced quantum memory system, respectively. The photon fluxes of the signal mode released from the memory system after passing through a filter system consisting of the polarizer and etalons with the external transmission of 85.6% is measured, thus the memory efficiency of 67 ± 1% is directly measured and real memory efficiency deducting the external transmission losses should be 78 ± 1%.

In the quantum memory, the fidelity $F={\{Tr[{({\hat{\rho }}_{1}^{1/2}{\hat{\rho }}_{2}{\hat{\rho }}_{1}^{1/2})}^{1/2}]\}}^{2}$, which describes the overlap of input states ${\hat{\rho }}_{1}$ and the states ${\hat{\rho }}_{2}$ released from the memory system, quantifies the performance of a quantum memory. Generally, if the average fidelities for a set of input coherent states within a Gaussian distribution surpass the classical benchmark fidelities, the quantum property outperforming classical systems can be verified^41,42^. Figure 2b presents the dependence of average fidelity on mean photon number $\overline{n}$ of the Gaussian distribution of input set of coherent states. Blue solid line represents the theoretical average fidelity, and the red dots are the corresponding experimentally measured results. The black dashed line is the corresponding classical benchmark fidelity. It shows that the average fidelity depends on the mean photon number of the Gaussian distribution of the input coherent state, and reaches 0.97 ± 0.01 for the input coherent state with the same mean photon number 0.60 of the Gaussian distribution of the input set of states, where the corresponding benchmark fidelity is 0.73. We can see that for a set of coherent states within the mean photon number range from $\overline{n}=0$ to $\overline{n}=8.0$, the average fidelities for each input state exceed its classical benchmark fidelity, thus the quantum property of the memory outperforming any classical memory is confirmed^35,36^ (see Supplementary Note 5 for details).

The memory efficiency and the average fidelity as functions of storage time are shown in Fig. 2c, where the average fidelity is determined by storing and releasing of various input coherent states with $\overline{n}=0.60$. Blue solid line and blue squares are the theoretical memory efficiency and the experimentally measured values, respectively; and red dotted line and red dots represent the theoretical average fidelity and experimentally measured results, respectively; the black dashed line stands for the classical benchmark fidelity. From blue solid line, the lifetime in the memory system with warm atomic cell of 1.2 μs is obtained, which is mainly limited by the magnetic noise. We can see that all average fidelities are higher than the classical benchmark fidelities within the lifetime of atoms, and the storage time can be chosen arbitrarily within the lifetime.

## Discussion

Due to the use of an optical cavity with the near-perfect temporal and spatial matching, the memory efficiency of 67 ± 1% and the excess noise close to QNL have been directly measured. For set of input coherent states within the mean photon number range from $\overline{n}=0$ to $\overline{n}=8.0$, the deterministic average fidelities have exceeded the benchmark fidelities. Thus, the memory has entered into the quantum region. Because the atomic cell is put in the single layer magnetic field shielding barrel in the present experiment, the memory lifetime is short at the scale of microseconds due to the influence of the residual magnetic field noise. If the magnetic field noise is further reduced by employing the multiple-layer structure, the lifetime must be obviously increased^43^. Alternatively, it has been demonstrated that the cell-wall anti-relaxation coating onto the inner surface of the cell may provide an effective approach to extend the memory lifetime of warm atom to the scale of milliseconds^5,44^. We believe that if above mentioned feasible techniques are applied in our system, a quantum memory with longer memory lifetime will be possibly demonstrated based on the cavity-enhanced warm atomic system. The presented approach is achievable on a variety of other physical platforms, such as in trapped ions^45–48^, superconductors^49–51^, solid states^52–55^ and optomechanics^56–60^.

For optical continuous-variable (CV) quantum information systems, the quantum information is encoded in the quadrature amplitudes and phases of optical signal modes, which have been used to implement quantum information protocols, such as quantum teleportation^61^, quantum dense coding^62^ and quantum dense metrology^63^. For the presented memory experiment of optical coherent state, the quantum information is encoded and stored in the quadrature amplitudes and phases of optical signal modes. Besides, photons carrying information in discrete variable (DV), such as its arbitrary polarization state, can also be stored in the presented system by replacing the Glan–Thompson polarizer with a polarization insensitive beam splitter^64^. Thus, the cavity-enhanced memory works equally well for both CV and DV quantum information because the cavity-enhanced quantum memory is a linear mapping technique^65^. Quantized states of light, such as squeezing^66^ and entanglement^67^ are kernel resources in quantum information science and technology, and the cavity-enhanced quantum memory system performs well enough to preserve quantum information of arbitrary quantized optical states. Due to the experimental simplicity of a warm atomic cell setup^68,69^, the presented system is robust and easily controlled, which is ready to be applied in some quantum information systems.

## Methods

### Experimental setup

The experimental setup for implementing the cavity-enhanced quantum memory is shown in Fig. 1b, and the experimental details are given (see Supplementary Note 1). A ^87^*Rb* atomic cell coated with 795 nm anti-reflection, which is placed in a magnetic shielding, is used as EIT medium of cavity-enhanced quantum memory system. An input signal mode with an optimized wave packet originally coming from Ti:sapphire laser is stored in an atomic cell inside the optical cavity. The time sequence of the cavity-enhanced quantum memory is given (see Supplementary Note 2). In this experiment, the photoreceiver detector D1 is applied to measure memory efficiency, which is theoretically analyzed in details (see Supplementary Note 3), and the excess noises can be analyzed from the BHD measurement (see Supplementary Note 4).

## Supplementary information

Supplementary Information

Supplementary figure

## Data Availability

The data that support the findings of this study are available within the paper and its Supplementary information. Additional data are available from the corresponding authors upon reasonable request.
