# Supplementary material for: High-performance cavity-enhanced quantum memory with warm atomic cell
Source: Nat Commun. 2022 May 2;13:2368. doi: 10.1038/s41467-022-30077-1 (PMC9061733; doi:10.1038/s41467-022-30077-1)
Supplement: Supplementary file 1 — Supplementary Information [file 41467_2022_30077_MOESM1_ESM.pdf]

SUPPLEMENTARY INFORMATION:
High-performance cavity-enhanced quantum memory with warm atomic cell

Lixia Ma[1,3], Xing Lei[1,3], Jieli Yan[1], Ruiyang Li[1], Ting Chai[1], Zhihui Yan[1,2*], Xiaojun Jia[1,2†], Changde Xie[1,2] & Kunchi Peng[1,2]

[1]State Key Laboratory of Quantum Optics and Quantum Optics Devices, Institute of Opto-Electronics, Shanxi University, Taiyuan 030006, P. R. China

[2]Collaborative Innovation Center of Extreme Optics, Shanxi University, Taiyuan 030006, P. R. China

[3]These authors contributed equally: Lixia Ma, Xing Lei.

[*]e-mail:zhyan@sxu.edu.cn

[†]e-mail:jiaxj@sxu.edu.cn

In the following, we provide additional information on high-performance cavity-enhanced quantum memory with warm atomic cell. In Supplementary Note 1, we discuss the experimental details of cavity-enhanced quantum memory. In Supplementary Note 2, the time sequence is shown. We study theoretical analyses of the efficiency, noise and fidelity of memory system in Supplementary Note 3, Note 4 and Note 5, respectively.

Supplementary Note 1 - Experimental details

The experimental setup for cavity-enhanced quantum memory is shown in Fig. 1 (b). A Ti:sapphire laser (Coherent MBR-110) pumped by green laser (Yuguang DPSS FG-VIIIB) outputs 3 W laser with coherent state, which is divided into three parts: the first part is injected into the cavity-enhanced quantum memory which is utilized as signal mode of quantum memory system, the second part is frequency shifted and power amplified which is used as control mode, and the last part is the local oscillation (LO) of balanced homodyne detector (BHD). An approximate exponential growth wave packet with the time constant of 50 ns to match the cavity-enhanced memory mode is used as the input signal mode, which is dynamically shaped in time by an electro-optical amplitude modulator (EOAM), and taken as the time reversed version of cavity-enhanced quantum memory to provide the optimum coupling. The control mode frequency shift of 6.8 GHz is realized by the electro-optical phase modulator (EOPM), and its power is amplified through the laser amplifier. The frequencies of both signal and control optical modes are red detuned by Δ_s=700 MHz (the detuning of signal optical mode from the transition between energy levels $\left|5S_{1/2}, F=1\right\rangle$ and $\left|5P_{1/2}, F'=1\right\rangle$) and Δ_c =700.5 MHz (the detuning of control optical

mode from the transition between energy levels $\left|5S_{1/2}, F=2\right\rangle$ and $\left|5P_{1/2}, F'=1\right\rangle$), respectively.

A ^{87}Rb atomic cell with 3 mm diameter and 10 mm length is filled with 10 torr of neon buffer gas. The atomic cell is put in the single layer magnetic field shielding barrel, and there is no magnetic field applied. However, along the axis of the magnetic field shielding barrel there are two small holes, from which the optical beams enter the barrel and pass through atomic cell, and the residual magnetic field direction along the axis of the magnetic field shielding barrel is used as the direction of the quantization axis. The electromagnetically-induced-transparency (EIT) medium with the bandwidth of about 2 MHz is employed in our memory system. The temperature of atomic cell is precisely controlled by the peltier with a temperature stabilization system and the accuracy of temperature control is within $0.01°\mathrm{C}$. The three different experimental conditions used for the noise analyses are three different optical depths, which can be controlled by changing the temperature of cell. The optical depths of 7, 15 and 90 correspond to the temperatures of $60°\mathrm{C}$, $70°\mathrm{C}$ and $95°\mathrm{C}$, respectively.

The atomic cell is placed in a bow-tie-type ring cavity with the length of 486 mm, and the diameters of both signal and control beams used inside the atomic cell are 514 μm, which can match the cavity mode near-perfectly. The finesse of an optical cavity, which is defined as the ratio between free spectral range and full width at half maximum (FWHM), is directly measured to be 17 for the present system, and thus the FWHM of the cavity resonance for the signal mode is 37 MHz. The input-output mirror transmission (T=0.30) is directly attained by measuring the transmitted and the incident laser power of the input-output mirror. The finesse depends on the transmission of the input-output mirror and the cavity loss according to $Finesse = \pi\sqrt{g_m}/\left(1-g_m\right)$, where loss parameter is $g_m = \sqrt{\left(1-T\right)\left(1-L\right)}$, here T is the transmission of input-output mirror and L is the cavity loss[1]. Using the measured finesse and the transmission, the cavity loss L=0.02 is calculated. The Pound-Drever-Hall (PDH) technique for cavity locking is implemented by using photoreceiver detector D2 to measure the transmitted optical mode after the Glan-Thompson polarizer P3.

The signal mode with horizontal polarization and control mode with vertical polarization are coupled into memory system with the polarizer P1. Only the signal mode with horizontal polarization can pass through the memory system with the polarizer P2, and etalons resonating merely with the signal mode; and the control mode polarizing at the vertical direction with different wavelength has been totally filtered out when the released signal mode arrives the detector. Input coherent optical mode and released mode are measured in the experiments. The measurement on the input

mode is performed on the input signal optical mode before entering the optical cavity. Then, the input optical signal and control modes are injected into the optical cavity including the atomic medium and the measurement on the released mode is performed on the optical pulse transmitted through the input-output mirror of the optical cavity. The photon fluxes of the input and released modes are measured by the photoreceiver detector. The quadrature amplitude (phase) components for the input and released optical states are measured by means of BHD, where the measurement bandwidth is 10.8 MHz. The released signal mode from the memory cavity travels through a polarizer and a series of etalons and finally arrives at the BHD with the external transmission of 85.6%, which mainly results from the etalon transmission of 86%, after the control mode is filtered out by the polarizer and etalons. The interference visibility between the signal mode and the LO mode on the BHD is 99%, and the quantum efficiency of photo-diode is 94%. Supplementary Fig. 1 is the reconstructed Wigner function of input (a) and released (b) states of cavity-enhanced quantum memory. The input and released Gaussian coherent states are proved by analyzing the density matrix intermediated Wigner function.

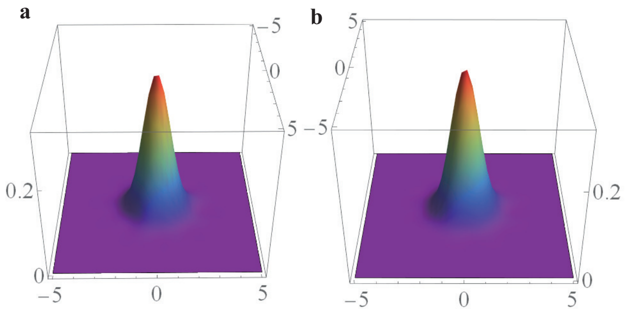

Supplementary Fig. 1. The reconstructed Wigner function of input (a) and released (b) states of cavity-enhanced quantum memory.

Supplementary Note 2 - Time sequence

Each time period includes a locking sequence (2 ms) and a memory sequence (10 μs). In locking sequence, the signal mode is turned on to be used for scanning or locking the cavity length and the relative phase between signal and LO optical modes in the BHD, where the relative phase between signal and LO optical modes is controlled by a Piezoelectric Transducer and locked by means of PDH technique. When their relative phase is locked at $k\pi$ $((1/2 + k)\pi)$, the quadrature

amplitude (phase) is recorded[1].

In the memory input and released signal modes are measured respectively at different time. Before quantum memory, the photon flux of an input signal mode is measured and shown in the red line of Fig. 2 (a). Quantum memory includes the writing $(t < 0)$, storage $(0 < t < T_0)$ and reading $(t > T_0)$ processes. The control mode is switched by a pair of acousto-optical modulators (AOMs) to implement the user-controlled quantum memory. In the quantum memory, the photon fluxes of the leakage and the released signal modes are measured and shown as the blue line in Fig. 2 (a). In the writing process, both the weak signal and the strong control modes interact with an atomic medium. In the storage process, the quantized state of light is stored and preserved in atom medium. The lifetime defined as a $1/e$ coherence time is 1.2 μs, and the storage time T_0=100 ns can be chosen by user within the lifetime. In the reading process, the optical mode is released from atomic medium and measured.

Supplementary Note 3 - Analysis of the memory efficiency

The photon fluxes of the input and released signal modes are directly measured with photoreceiver detector. The photon flux of input signal mode is firstly measured in the case without the optical cavity and atomic medium, then the atomic cell is placed in the optical cavity and the photon flux of the signal mode released from the input-output mirror of cavity is measured. For analyzing the memory efficiency exactly, the coherent state with mean photon number of 2.9×10^6 is employed in this experiment. All parameter values are experimentally reachable to provide direct references for experimental system design, where the effective light-atom coupling constant κ is 59 MHz, the spin wave decoherence rate γ_0 is 420 kHz, and the Doppler broadening is 500 MHz. The control optical mode with the power of 2.0 mW enters the cavity to implement the memory interaction, when the signal mode is on resonance with the cavity. Supplementary Fig. 2 shows that the dependence of memory efficiency on the transmission of the input-output mirror T and the cavity loss L for the storage time of 100 ns. From it, we can see that the smaller cavity loss is, the better the memory efficiency is. The black star depicts the experimental result of $\eta(T_0)$ =78$\pm$1% where the transmission of the input-output mirror T is 0.30 and the cavity loss L is about 0.02. We measured the function of the memory efficiency on the control power in Supplementary Fig. 3, which is well fitted to the theoretical result of cavity-enhanced EIT memory without considering the influence of four-wave-mixing (FWM) amplification.

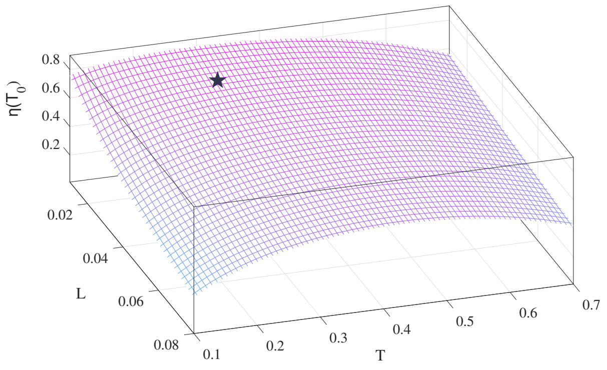

Supplementary Fig. 2. The dependence of memory efficiency $\eta(\mathrm{T}_0)$ on the transmission of the input-output mirror T and the cavity loss L. The black star corresponds the experimentally measured result.

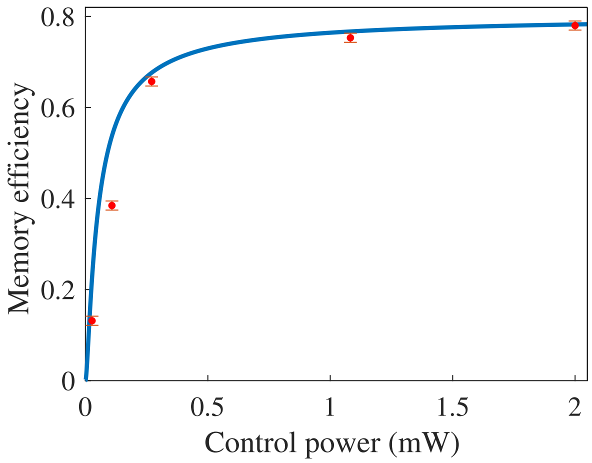

Supplementary Fig. 3. The memory efficiency vs the control power. The blue line is the theoretical curve. The red dots correspond the experimentally measured results. Error bars represent ± 1 standard error are obtained with the statistics of the measured photon numbers.

Supplementary Note 4 - Analysis of the memory noise

By adjusting the length of the cavity in the experiment, the signal mode is resonant with the optical cavity, while the FWM optical mode is off resonant. Thus, the memory interaction is enhanced by resonating the signal mode and the scattered FWM mode is suppressed in the cavity. A sketch shows the different frequencies with respect to the spectrum of the cavity in Supplementary

Fig. 4. Based on the equations for the interference of multiple optical modes inside a cavity, the reflected power spectrum of cavity is obtained, that is, $P_{refl} = P_{in}(|R - g(\nu)|^2)/(R|1 - g(\nu)|^2)$, where P_{in} and P_{refl} are the input and the reflected power of cavity, respectively; R is the reflectivity of input-output mirror; $g(\nu)$ is a complex number which describes the total effect of the cavity during one round trip[1]. The general expression for the noise suppression factor is $S = (1 - \mu_s e^{i\varphi_s}) / (1 - \mu_f e^{-i\varphi_f})$, where the $\mu_{f(s)}$ and $\varphi_{f(s)}$ are the cavity-round-trip amplitude transmissions and phases at the FWM (signal) mode, respectively[2]. In our system, noise suppression factor S is 0.35 with the phases at FWM and signal modes of 0.4π and 0, respectively[3].

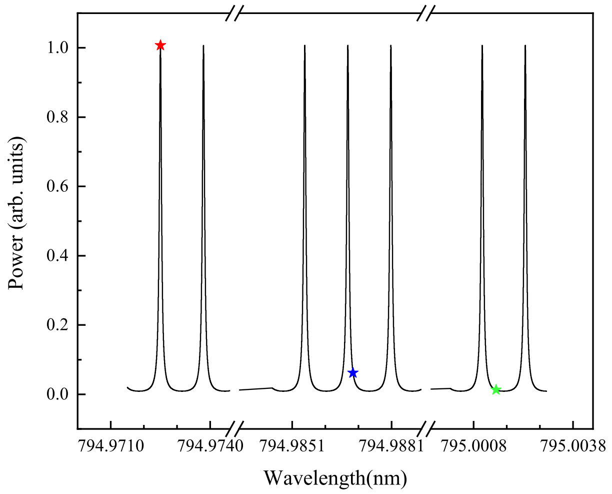

Supplementary Fig. 4. The spectrum of the cavity on the different frequencies. The red, blue and green stars correspond the experimentally measured results of signal, control and FWM modes, respectively.

The quantum noise of a quadrature component for an ideal coherent state should equal to the quantum noise limit (QNL). Due to the existence of the unavoidable excess noises in the experiment, the noise values of the measured states are always higher than the QNL. The laser used to create the signal mode is a coherent state at QNL level. The input mode of quantum memory is measured on the transmitted pulse in the situation without atomic medium. The cavity with atomic medium is placed in the optical path of the input mode and then the released mode transmitted from the input-output mirror of the optical cavity including the atomic medium is measured. All the possible extra noises on the signal mode introduced by the memory are retained

and measured with the BHD. Their quadrature amplitude (phase) values are measured by BHD and their fluctuations are obtained by means of the statistics of the integrated temporal modes[4], and the noise characterizations of the released modes are normalized to the QNL. The excess noises of quadrature amplitudes (phases) normalized to the QNL for the released states with three different memory efficiencies of 51%, 60% and 67% are 1.026±0.02 (1.025±0.02), 1.025±0.02 (1.028±0.02) and 1.025±0.02 (1.027±0.02), respectively. We can see that the excess noises of the quantum memory are almost independent of the memory efficiencies, that means the excess noise is effectively suppressed by the optical cavity. Besides the noise suppression factor S, the parameter $\mu_1 = \overline{n}_{noise}/\eta_{total}$ is a convenient metric to determine the performance of noise suppression[3]. When the input state is vacuum input, a direct measurement of the noise floor give $\overline{n}_{noise} = 0.10$ photons per control pulse in the readout time and the memory efficiency is $\eta_{total} = 67\%$ for our system the calculated parameter is $\mu_1 = 0.15$.

Supplementary Note 5 - Analysis of the memory fidelity

The classical benchmark fidelities for storing and releasing a number of input coherent states with the Gaussian distribution have been derived, as $F_{class} = (1+\overline{n})/(1+2\overline{n})$, where $\overline{n}$ is the mean photon number of Gaussian distribution[5].

In continuous-variable (CV) quantum information, the quadrature components are used to encode information, and quantum state can be totally expressed by the covariance matrices with the mean values of quadrature components. For a given CV input (released) state with the mean amplitudes $\alpha_{1(2)} = \begin{pmatrix} \alpha_{x1(2)} \\ \alpha_{p1(2)} \end{pmatrix}$ and the covariance matrices $A_{1(2)} = 4 \begin{pmatrix} \langle \Delta^2(\hat{x}) \rangle_{1(2)} & 0 \\ 0 & \langle \Delta^2(\hat{p}) \rangle_{1(2)} \end{pmatrix}$, the single-shot fidelity can be given by

$$F_S = \frac{2}{\sqrt{\Delta + \sigma} - \sqrt{\sigma}} \exp[-\varepsilon^T (A_1 + A_2)^{-1} \varepsilon], \tag{1}$$

where $\varepsilon = \alpha_2 - \alpha_1$, $\Delta = \det(A_1 + A_2)$, $\sigma = (\det A_1 - 1)(\det A_2 - 1)$[6–8]. For a set of coherent states with a Gaussian distribution of displacements with mean photon number $\overline{n}$, the average fidelity is calculated by integrating the single-shot fidelity, which is

$$F_A = \frac{\sqrt{2}}{\sqrt{1 + 2\sqrt{\Delta^2(\hat{x}_2)} + 2\overline{n}(1 - |\overline{x}_2/\overline{x}_1|)^2}} \times \frac{\sqrt{2}}{\sqrt{1 + 2\sqrt{\Delta^2(\hat{p}_2)} + 2\overline{n}(1 - |\overline{p}_2/\overline{p}_1|)^2}}, \tag{2}$$

where $\overline{x}_{1(2)}$ and $\overline{p}_{1(2)}$ are the mean values of amplitude and phase quadratures for input (output) coherent states, respectively[9]. The memory experiment is implemented by using a Gaussian distribution set of input coherent states, whose amplitudes and phases are varied. The fidelities of 5 different coherent states with a Gaussian distribution of displacement with mean photon numbers of 0.6, 2.0, 4.0, 6.0, 8.0 in vacuum units respectively are measured. For each coherent state with a given mean photon number of the Gaussian distribution the fidelity measurement is performed three times at three different phases of 0, $\pi/4$, $\pi/2$ and then the average fidelity for different phases is obtained. To obtain a reliable average fidelity memory experiment is performed 10000 times on a given coherent state with a selected phase, and then the average result is taken.

References

[1] Bachor, H. A. & Ralph, T. C. *A Guide to Experiments in Quantum Optics* (Wiley-vch, Weinheim, Berlin, 2004).

[2] Nunn, J., Munns, J. H. D., Thomas, S., Kaczmarek, K. T., Qiu, C., Feizpour, A., Poem, E., Brecht, B., Saunders, D. J., Ledingham, P. M., Reddy, D. V., Raymer, M. G. & Walmsley, I. A. Theory of noise suppression in Λ-type quantum memories by means of a cavity. *Phys. Rev. A* **96**, 012338 (2017).

[3] Saunders, D. J., Munns, J. H. D., Champion, T. F. M., Qiu, C., Kaczmarek, K. T., Poem, E., Ledingham, P. M., Walmsley, I. A. & Nunn, J. Cavity-Enhanced room-temperature broadband Raman memory. *Phys. Rev. Lett.* **116**, 090501 (2016).

[4] Honda, K., Akamatsu, D., Arikawa, M., Yokoi, Y., Akiba, K., Nagatsuka, S., Tanimura, T., Furusawa, A. & Kozuma, M. Storage and retrieval of a squeezed vacuum. *Phys. Rev. Lett.* **100**, 093601 (2008).

[5] Hammerer, K., Wolf, M. M., Polzik, E. S. & Cirac. J. I. Quantum benchmark for storage and transmission of coherent states. *Phys. Rev. Lett.* **94,** 150503 (2005).

[6] Nha, H. & Carmichael, H. J. Distinguishing two single-mode gaussian states by homodyne detection: an information-theoretic approach. *Phys. Rev. A* **71**, 032336 (2005).

[7] Scutaru, H. Fidelity for displaced squeezed thermal states and the oscillator semigroup. *J. Phys. A: Math. Gen.* **31**, 3659 (1998).

[8] Su, X.-L., Hao, S.-H., Deng, X.-W., Ma, L.-Y., Wang, M.-H., Jia, X.-J., Xie, C.-D. & Peng, K.-C. Gate

sequence for continuous variable one-way quantum computation. *Nat. Commun.* **4**, 2828 (2013).

9 Krauter, H., Salart, D., Muschik, C. A., Petersen, J. M., Shen, H., Fernholz, T. & Polzik, E. S. Deterministic quantum teleportation between distant atomic objects. *Nat. Phys.* **9,** 400-404 (2013).
