# Supplementary figures and images for: High-performance cavity-enhanced quantum memory with warm atomic cell
Source: Nat Commun. 2022 May 2;13:2368. doi: 10.1038/s41467-022-30077-1 (PMC9061733; doi:10.1038/s41467-022-30077-1)

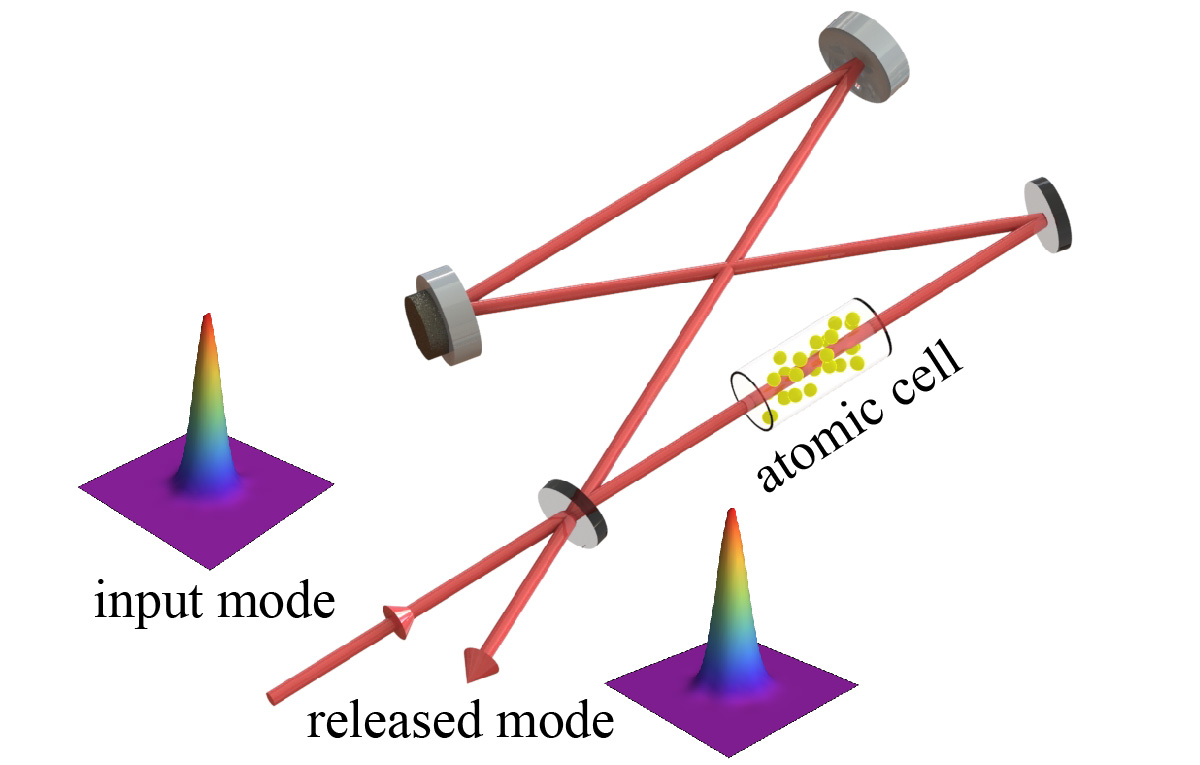

Supplement: Supplementary file 2 — Supplementary figure [file 41467_2022_30077_MOESM2_ESM.jpg]
